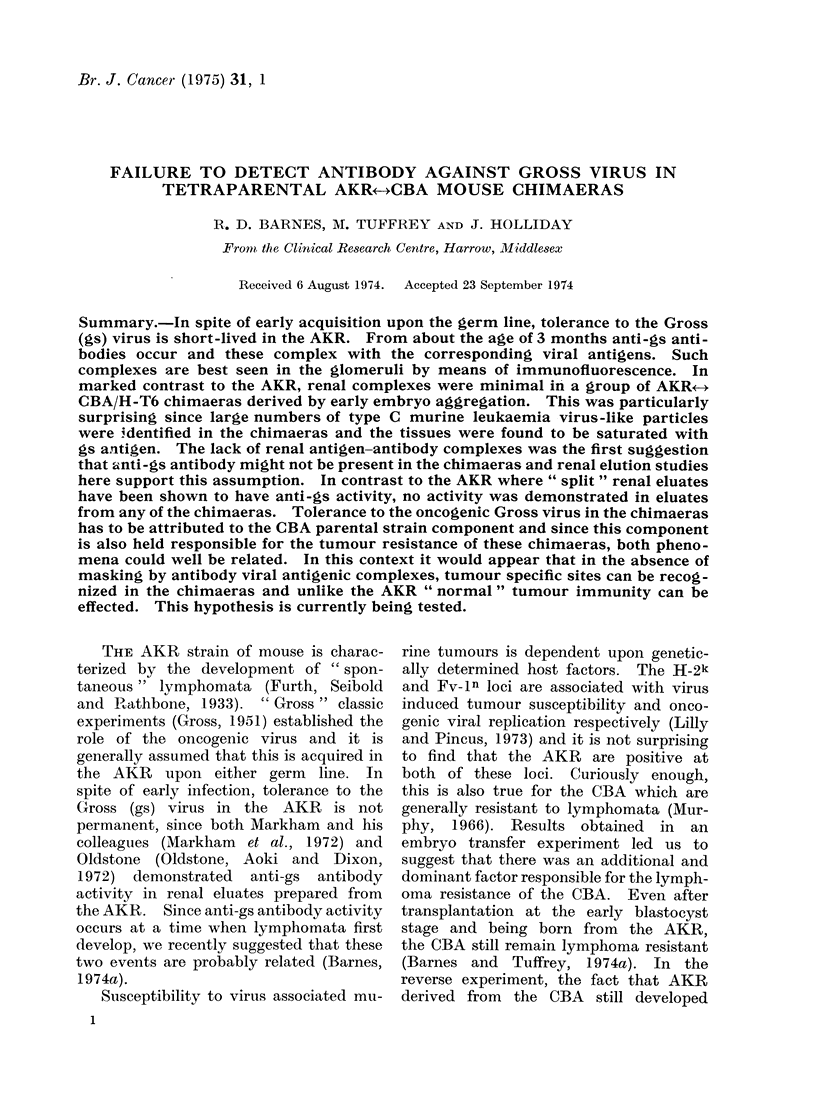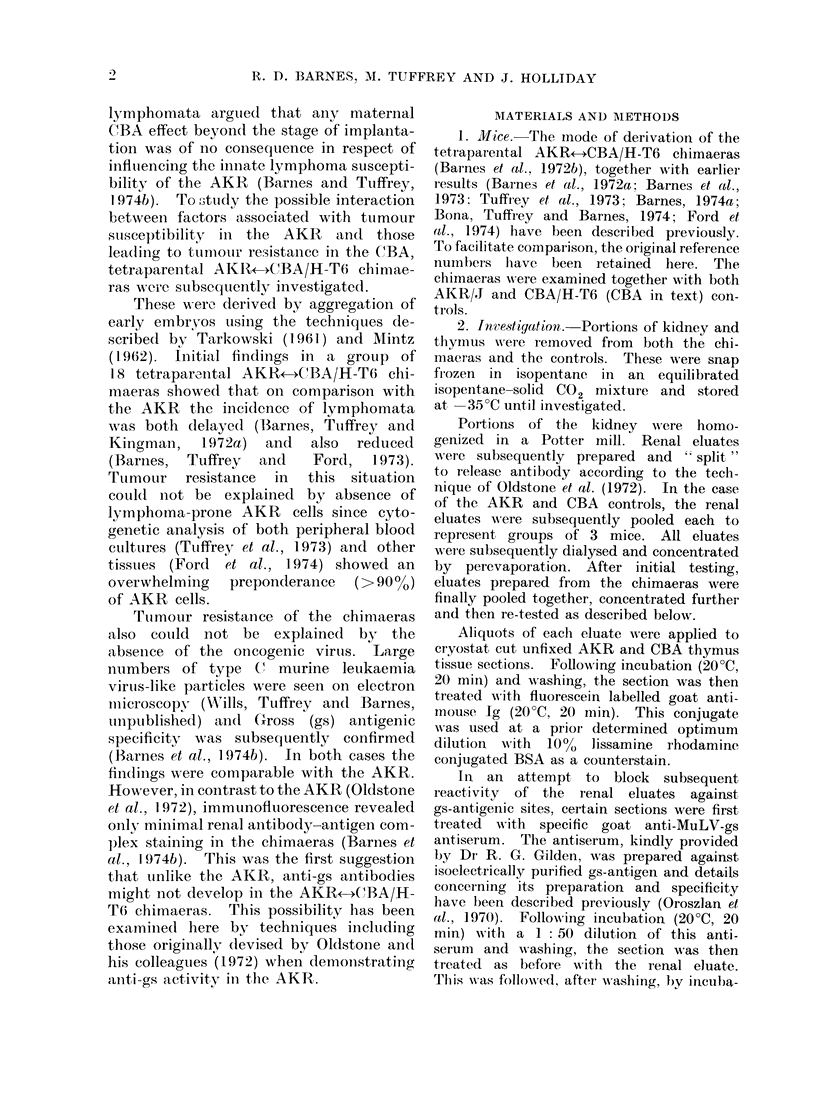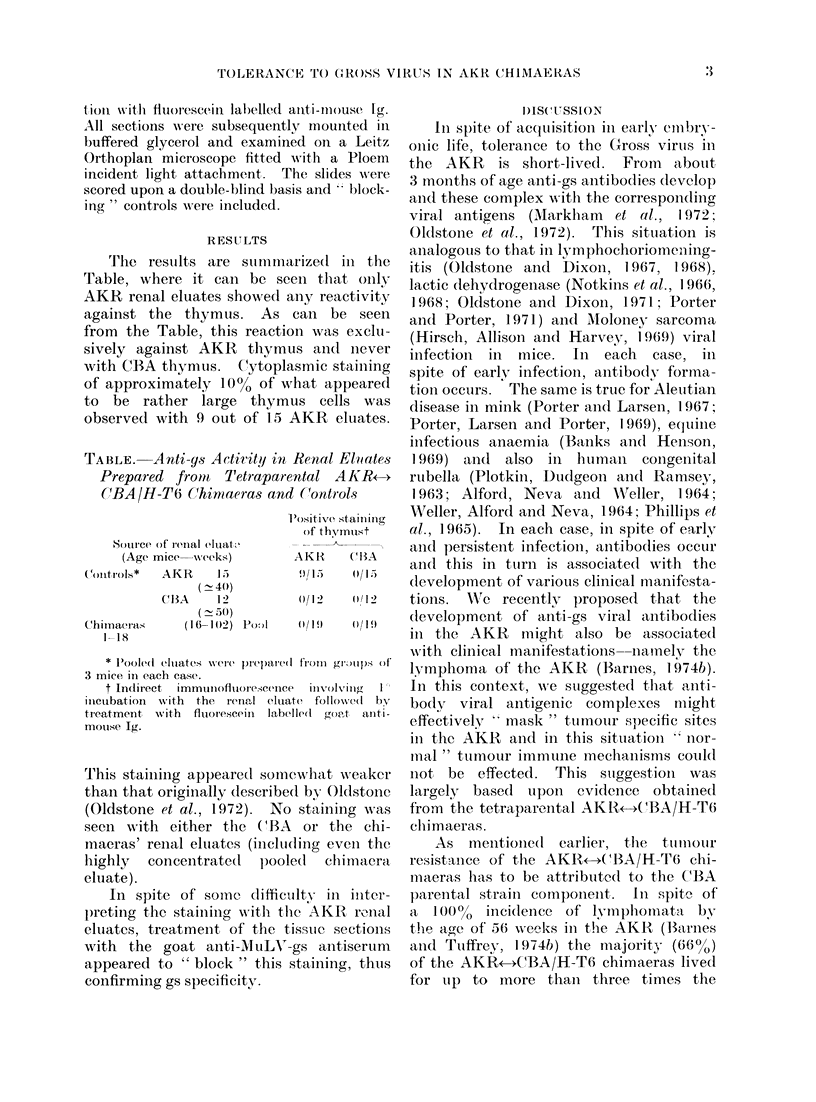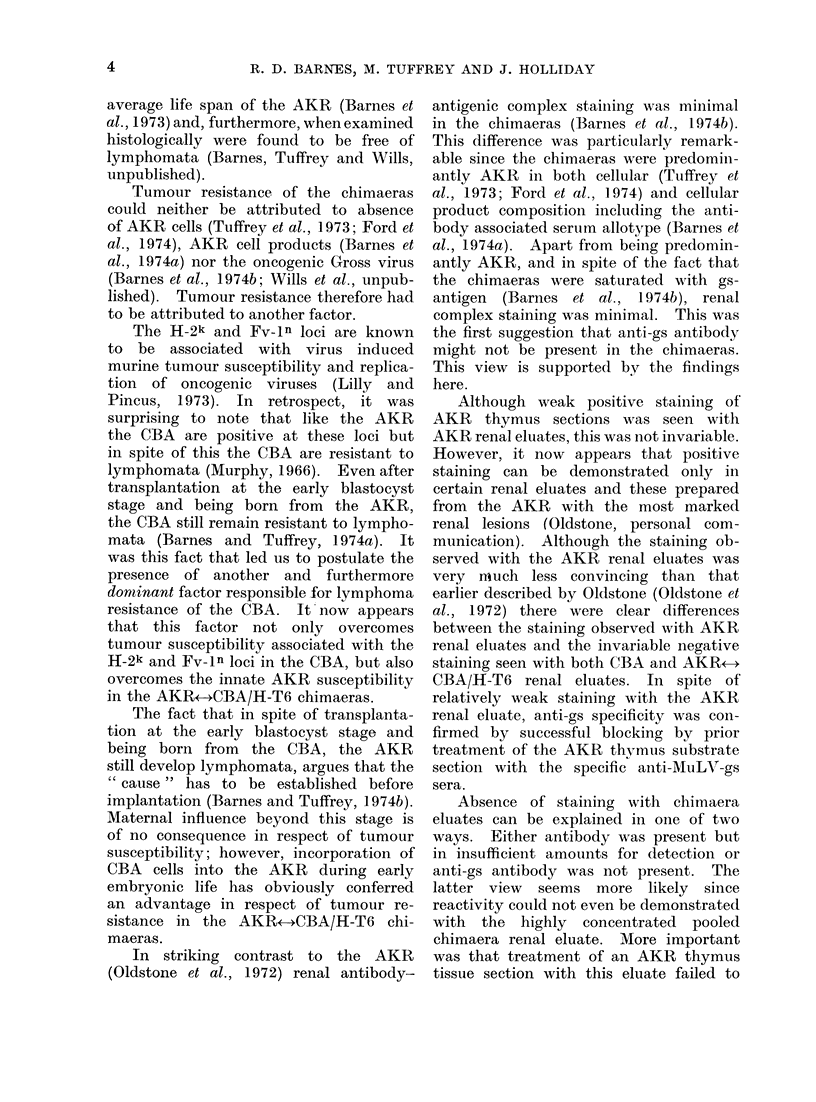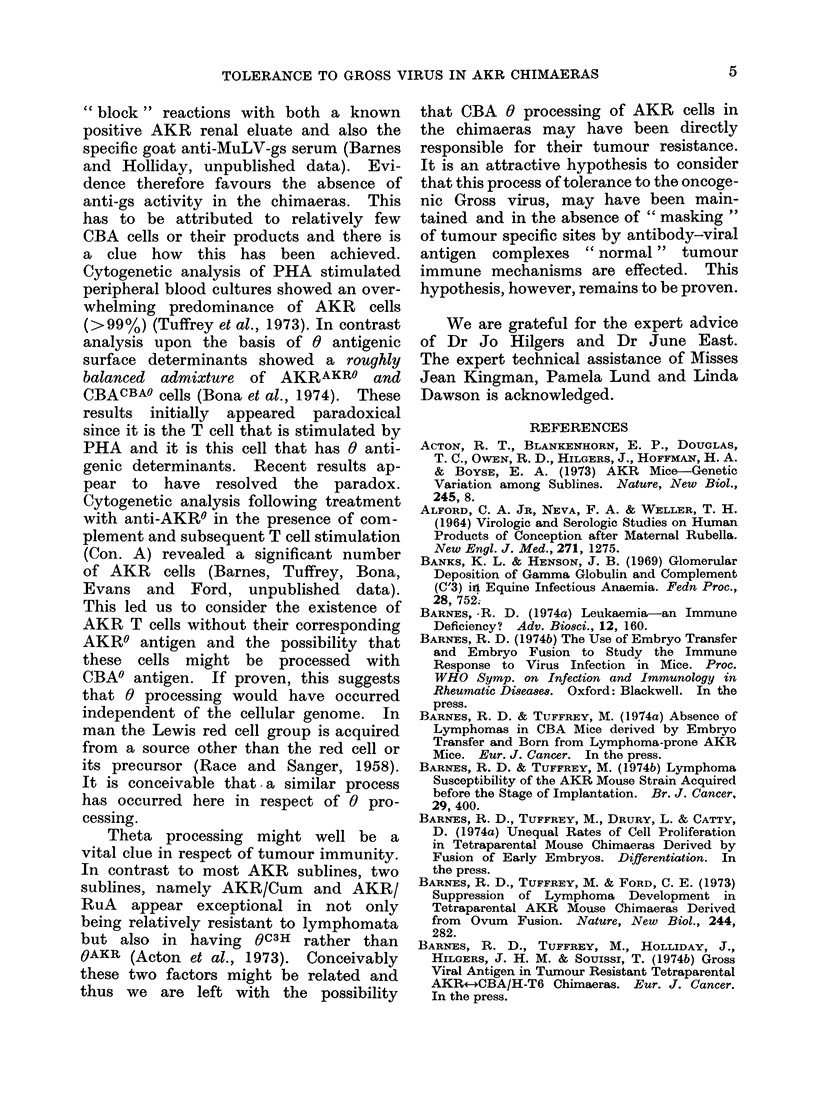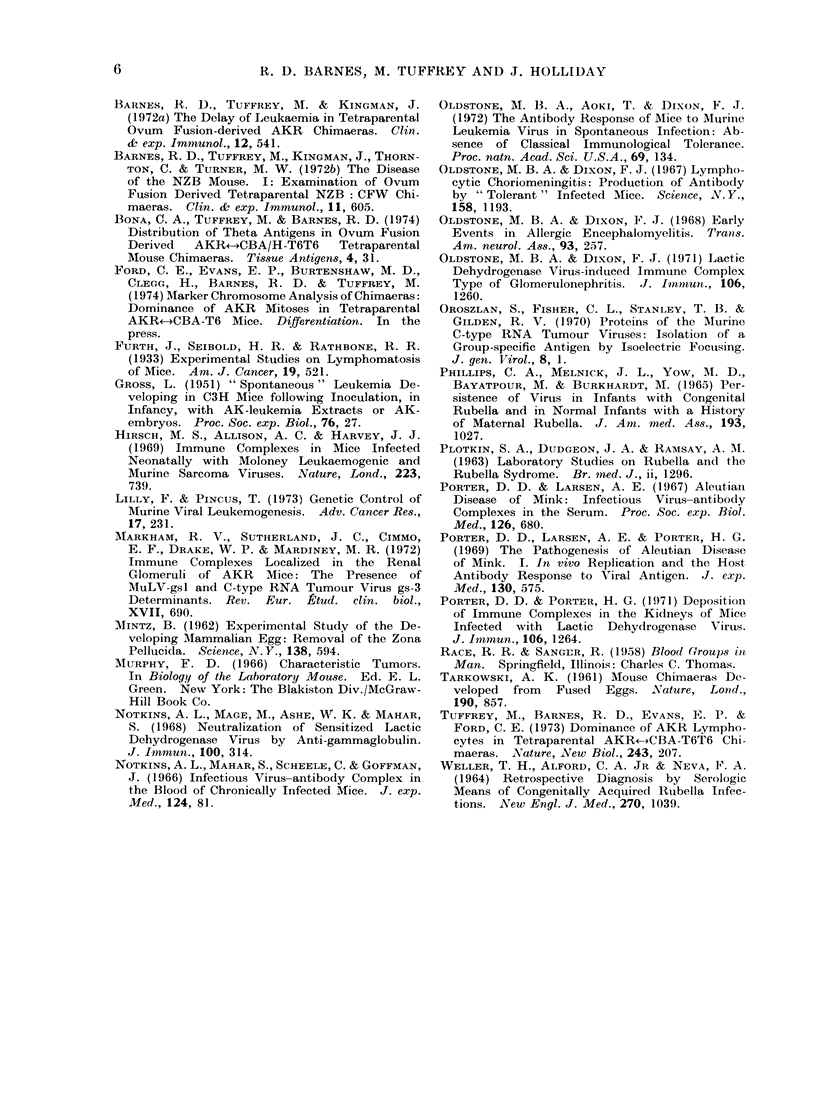# Failure to detect antibody against Gross virus in tetraparental AKR reversible CBA mouse chimaeras.

**DOI:** 10.1038/bjc.1975.1

**Published:** 1975-01

**Authors:** R. D. Barnes, M. Tuffrey, J. Holliday

## Abstract

In spite of early acquisition upon the germ line, tolerance to the Gross (gs) virus is short-lived in the AKR. From about the age of 3 months anti-gs antibodies occur and these complex with the corresponding viral antigens. Such complexes are best seen in the glomeruli by means of immunofluorescence. In marked contrast to the AKR, renal complexes were minimal in a group of AKR reversible CBA/H-T6 chimaeras derived by early embryo aggregation. This was particularly surprising since large numbers of type C murine leukaemia virus-like particles were identified in the chimaeras and the tissues were found to be saturated with gs antigen. The lack of renal antigen-antibody complexes was the first suggestion that anti-gs antibody might not be present in the chimaeras and renal elution studies here support this assumption. In contrast to the AKR where "split " renal eluates have been shown to have anti-gs activity, no activity was demonstrated in eluates from any of the chimaras. Tolerance to the oncogenic Gross virus in the chimaeras has to be attributed to the CBA parental strain component and since this component is also held responsible for the tumour resistance of these chimaeras, both phenomena could well be related. In this context it would appear that in the absence of masking by antibody viral antigenic complexes, tumour specific sites can be recognized in the chimaeras and unlike the AKR "normal" tumour immunity can be effected. This hypothesis is currently bei-ng tested.


*Br. J. Cancer* (1975) **31**, 1

### FAILURE TO DETECT ANTIBODY AGAINST GROSS VIRUS IN TETRAPARENTAL AKR↔CBA MOUSE CHIMAERAS

R. D. BARNES, M. TUFFREY AND J. HOLLIDAY

*From the Clinical Research Centre, Harrow, Middlesex*

Received 6 August 1974. Accepted 23 September 1974

**Summary.—In spite of early acquisition upon the germ line, tolerance to the Gross (gs) virus is short-lived in the AKR. From about the age of 3 months anti-gs antibodies occur and these complex with the corresponding viral antigens. Such complexes are best seen in the glomeruli by means of immunofluorescence. In marked contrast to the AKR, renal complexes were minimal in a group of AKR↔ CBA/H-T6 chimaeras derived by early embryo aggregation. This was particularly surprising since large numbers of type C murine leukaemia virus-like particles were identified in the chimaeras and the tissues were found to be saturated with gs antigen. The lack of renal antigen–antibody complexes was the first suggestion that anti-gs antibody might not be present in the chimaeras and renal elution studies here support this assumption. In contrast to the AKR where " split " renal eluates have been shown to have anti-gs activity, no activity was demonstrated in eluates from any of the chimaeras. Tolerance to the oncogenic Gross virus in the chimaeras has to be attributed to the CBA parental strain component and since this component is also held responsible for the tumour resistance of these chimaeras, both phenomena could well be related. In this context it would appear that in the absence of masking by antibody viral antigenic complexes, tumour specific sites can be recognized in the chimaeras and unlike the AKR " normal " tumour immunity can be effected. This hypothesis is currently being tested.**

THE AKR strain of mouse is characterized by the development of " spontaneous " lymphomata (Furth, Seibold and Rathbone, 1933). " Gross " classic experiments (Gross, 1951) established the role of the oncogenic virus and it is generally assumed that this is acquired in the AKR upon either germ line. In spite of early infection, tolerance to the Gross (gs) virus in the AKR is not permanent, since both Markham and his colleagues (Markham *et al.*, 1972) and Oldstone (Oldstone, Aoki and Dixon, 1972) demonstrated anti-gs antibody activity in renal eluates prepared from the AKR. Since anti-gs antibody activity occurs at a time when lymphomata first develop, we recently suggested that these two events are probably related (Barnes, 1974*a*).

Susceptibility to virus associated murine tumours is dependent upon genetically determined host factors. The $H\text{-}2^k$ and $Fv\text{-}1^n$ loci are associated with virus induced tumour susceptibility and oncogenic viral replication respectively (Lilly and Pincus, 1973) and it is not surprising to find that the AKR are positive at both of these loci. Curiously enough, this is also true for the CBA which are generally resistant to lymphomata (Murphy, 1966). Results obtained in an embryo transfer experiment led us to suggest that there was an additional and dominant factor responsible for the lymphoma resistance of the CBA. Even after transplantation at the early blastocyst stage and being born from the AKR, the CBA still remain lymphoma resistant (Barnes and Tuffrey, 1974*a*). In the reverse experiment, the fact that AKR derived from the CBA still developed lymphomata argued that any maternal CBA effect beyond the stage of implantation was of no consequence in respect of influencing the innate lymphoma susceptibility of the AKR (Barnes and Tuffrey, 1974*b*). To study the possible interaction between factors associated with tumour susceptibility in the AKR and those leading to tumour resistance in the CBA, tetraparental AKR↔CBA/H-T6 chimaeras were subsequently investigated.

These were derived by aggregation of early embryos using the techniques described by Tarkowski (1961) and Mintz (1962). Initial findings in a group of 18 tetraparental AKR↔CBA/H-T6 chimaeras showed that on comparison with the AKR the incidence of lymphomata was both delayed (Barnes, Tuffrey and Kingman, 1972*a*) and also reduced (Barnes, Tuffrey and Ford, 1973). Tumour resistance in this situation could not be explained by absence of lymphoma-prone AKR cells since cytogenetic analysis of both peripheral blood cultures (Tuffrey *et al.*, 1973) and other tissues (Ford *et al.*, 1974) showed an overwhelming preponderance (>90%) of AKR cells.

Tumour resistance of the chimaeras also could not be explained by the absence of the oncogenic virus. Large numbers of type C murine leukaemia virus-like particles were seen on electron microscopy (Wills, Tuffrey and Barnes, unpublished) and Gross (gs) antigenic specificity was subsequently confirmed (Barnes *et al.*, 1974*b*). In both cases the findings were comparable with the AKR. However, in contrast to the AKR (Oldstone *et al.*, 1972), immunofluorescence revealed only minimal renal antibody–antigen complex staining in the chimaeras (Barnes *et al.*, 1974*b*). This was the first suggestion that unlike the AKR, anti-gs antibodies might not develop in the AKR↔CBA/H-T6 chimaeras. This possibility has been examined here by techniques including those originally devised by Oldstone and his colleagues (1972) when demonstrating anti-gs activity in the AKR.

### MATERIALS AND METHODS

1. *Mice.*—The mode of derivation of the tetraparental AKR↔CBA/H-T6 chimaeras (Barnes *et al.*, 1972*b*), together with earlier results (Barnes *et al.*, 1972*a*; Barnes *et al.*, 1973; Tuffrey *et al.*, 1973; Barnes, 1974*a*; Bona, Tuffrey and Barnes, 1974; Ford *et al.*, 1974) have been described previously. To facilitate comparison, the original reference numbers have been retained here. The chimaeras were examined together with both AKR/J and CBA/H-T6 (CBA in text) controls.

2. *Investigation.*—Portions of kidney and thymus were removed from both the chimaeras and the controls. These were snap frozen in isopentane in an equilibrated isopentane–solid $CO_2$ mixture and stored at −35°C until investigated.

Portions of the kidney were homogenized in a Potter mill. Renal eluates were subsequently prepared and "split" to release antibody according to the technique of Oldstone *et al.* (1972). In the case of the AKR and CBA controls, the renal eluates were subsequently pooled each to represent groups of 3 mice. All eluates were subsequently dialysed and concentrated by perevaporation. After initial testing, eluates prepared from the chimaeras were finally pooled together, concentrated further and then re-tested as described below.

Aliquots of each eluate were applied to cryostat cut unfixed AKR and CBA thymus tissue sections. Following incubation (20°C, 20 min) and washing, the section was then treated with fluorescein labelled goat anti-mouse Ig (20°C, 20 min). This conjugate was used at a prior determined optimum dilution with 10% lissamine rhodamine conjugated BSA as a counterstain.

In an attempt to block subsequent reactivity of the renal eluates against gs-antigenic sites, certain sections were first treated with specific goat anti-MuLV-gs antiserum. The antiserum, kindly provided by Dr R. G. Gilden, was prepared against isoelectrically purified gs-antigen and details concerning its preparation and specificity have been described previously (Oroszlan *et al.*, 1970). Following incubation (20°C, 20 min) with a 1 : 50 dilution of this antiserum and washing, the section was then treated as before with the renal eluate. This was followed, after washing, by incubation with fluorescein labelled anti-mouse Ig. All sections were subsequently mounted in buffered glycerol and examined on a Leitz Orthoplan microscope fitted with a Ploem incident light attachment. The slides were scored upon a double-blind basis and "blocking" controls were included.

### RESULTS

The results are summarized in the Table, where it can be seen that only AKR renal eluates showed any reactivity against the thymus. As can be seen from the Table, this reaction was exclusively against AKR thymus and never with CBA thymus. Cytoplasmic staining of approximately 10% of what appeared to be rather large thymus cells was observed with 9 out of 15 AKR eluates.

TABLE.—*Anti-gs Activity in Renal Eluates Prepared from Tetraparental AKR↔CBA/H-T6 Chimaeras and Controls*

| Source of renal eluate (Age mice—weeks) | | | | Positive staining of thymus† AKR | CBA |
|---|---|---|---|---|---|
| Controls* | AKR | 15 (≃40) | | 9/15 | 0/15 |
| | CBA | 12 (≃50) | | 0/12 | 0/12 |
| Chimaeras 1–18 | | (16–102) | Pool | 0/19 | 0/19 |

* Pooled eluates were prepared from groups of 3 mice in each case.

† Indirect immunofluorescence involving 1° incubation with the renal eluate followed by treatment with fluorescein labelled goat anti-mouse Ig.

This staining appeared somewhat weaker than that originally described by Oldstone (Oldstone *et al.*, 1972). No staining was seen with either the CBA or the chimaeras' renal eluates (including even the highly concentrated pooled chimaera eluate).

In spite of some difficulty in interpreting the staining with the AKR renal eluates, treatment of the tissue sections with the goat anti-MuLV-gs antiserum appeared to "block" this staining, thus confirming gs specificity.

### DISCUSSION

In spite of acquisition in early embryonic life, tolerance to the Gross virus in the AKR is short-lived. From about 3 months of age anti-gs antibodies develop and these complex with the corresponding viral antigens (Markham *et al.*, 1972; Oldstone *et al.*, 1972). This situation is analogous to that in lymphochoriomeningitis (Oldstone and Dixon, 1967, 1968), lactic dehydrogenase (Notkins *et al.*, 1966, 1968; Oldstone and Dixon, 1971; Porter and Porter, 1971) and Moloney sarcoma (Hirsch, Allison and Harvey, 1969) viral infection in mice. In each case, in spite of early infection, antibody formation occurs. The same is true for Aleutian disease in mink (Porter and Larsen, 1967; Porter, Larsen and Porter, 1969), equine infectious anaemia (Banks and Henson, 1969) and also in human congenital rubella (Plotkin, Dudgeon and Ramsey, 1963; Alford, Neva and Weller, 1964; Weller, Alford and Neva, 1964; Phillips *et al.*, 1965). In each case, in spite of early and persistent infection, antibodies occur and this in turn is associated with the development of various clinical manifestations. We recently proposed that the development of anti-gs viral antibodies in the AKR might also be associated with clinical manifestations—namely the lymphoma of the AKR (Barnes, 1974*b*). In this context, we suggested that antibody viral antigenic complexes might effectively "mask" tumour specific sites in the AKR and in this situation "normal" tumour immune mechanisms could not be effected. This suggestion was largely based upon evidence obtained from the tetraparental AKR↔CBA/H-T6 chimaeras.

As mentioned earlier, the tumour resistance of the AKR↔CBA/H-T6 chimaeras has to be attributed to the CBA parental strain component. In spite of a 100% incidence of lymphomata by the age of 56 weeks in the AKR (Barnes and Tuffrey, 1974*b*) the majority (66%) of the AKR↔CBA/H-T6 chimaeras lived for up to more than three times the average life span of the AKR (Barnes *et al.*, 1973) and, furthermore, when examined histologically were found to be free of lymphomata (Barnes, Tuffrey and Wills, unpublished).

Tumour resistance of the chimaeras could neither be attributed to absence of AKR cells (Tuffrey *et al.*, 1973; Ford *et al.*, 1974), AKR cell products (Barnes *et al.*, 1974*a*) nor the oncogenic Gross virus (Barnes *et al.*, 1974*b*; Wills *et al.*, unpublished). Tumour resistance therefore had to be attributed to another factor.

The H-$2^k$ and Fv-$1^n$ loci are known to be associated with virus induced murine tumour susceptibility and replication of oncogenic viruses (Lilly and Pincus, 1973). In retrospect, it was surprising to note that like the AKR the CBA are positive at these loci but in spite of this the CBA are resistant to lymphomata (Murphy, 1966). Even after transplantation at the early blastocyst stage and being born from the AKR, the CBA still remain resistant to lymphomata (Barnes and Tuffrey, 1974*a*). It was this fact that led us to postulate the presence of another and furthermore *dominant* factor responsible for lymphoma resistance of the CBA. It now appears that this factor not only overcomes tumour susceptibility associated with the H-$2^k$ and Fv-$1^n$ loci in the CBA, but also overcomes the innate AKR susceptibility in the AKR↔CBA/H-T6 chimaeras.

The fact that in spite of transplantation at the early blastocyst stage and being born from the CBA, the AKR still develop lymphomata, argues that the " cause " has to be established before implantation (Barnes and Tuffrey, 1974*b*). Maternal influence beyond this stage is of no consequence in respect of tumour susceptibility; however, incorporation of CBA cells into the AKR during early embryonic life has obviously conferred an advantage in respect of tumour resistance in the AKR↔CBA/H-T6 chimaeras.

In striking contrast to the AKR (Oldstone *et al.*, 1972) renal antibody–antigenic complex staining was minimal in the chimaeras (Barnes *et al.*, 1974*b*). This difference was particularly remarkable since the chimaeras were predominantly AKR in both cellular (Tuffrey *et al.*, 1973; Ford *et al.*, 1974) and cellular product composition including the antibody associated serum allotype (Barnes *et al.*, 1974*a*). Apart from being predominantly AKR, and in spite of the fact that the chimaeras were saturated with gs-antigen (Barnes *et al.*, 1974*b*), renal complex staining was minimal. This was the first suggestion that anti-gs antibody might not be present in the chimaeras. This view is supported by the findings here.

Although weak positive staining of AKR thymus sections was seen with AKR renal eluates, this was not invariable. However, it now appears that positive staining can be demonstrated only in certain renal eluates and these prepared from the AKR with the most marked renal lesions (Oldstone, personal communication). Although the staining observed with the AKR renal eluates was very much less convincing than that earlier described by Oldstone (Oldstone *et al.*, 1972) there were clear differences between the staining observed with AKR renal eluates and the invariable negative staining seen with both CBA and AKR↔CBA/H-T6 renal eluates. In spite of relatively weak staining with the AKR renal eluate, anti-gs specificity was confirmed by successful blocking by prior treatment of the AKR thymus substrate section with the specific anti-MuLV-gs sera.

Absence of staining with chimaera eluates can be explained in one of two ways. Either antibody was present but in insufficient amounts for detection or anti-gs antibody was not present. The latter view seems more likely since reactivity could not even be demonstrated with the highly concentrated pooled chimaera renal eluate. More important was that treatment of an AKR thymus tissue section with this eluate failed to

"block" reactions with both a known positive AKR renal eluate and also the specific goat anti-MuLV-gs serum (Barnes and Holliday, unpublished data). Evidence therefore favours the absence of anti-gs activity in the chimaeras. This has to be attributed to relatively few CBA cells or their products and there is a clue how this has been achieved. Cytogenetic analysis of PHA stimulated peripheral blood cultures showed an overwhelming predominance of AKR cells (>99%) (Tuffrey *et al.*, 1973). In contrast analysis upon the basis of $\theta$ antigenic surface determinants showed a *roughly balanced admixture* of $AKR^{AKR\theta}$ *and* $CBA^{CBA\theta}$ cells (Bona *et al.*, 1974). These results initially appeared paradoxical since it is the T cell that is stimulated by PHA and it is this cell that has $\theta$ antigenic determinants. Recent results appear to have resolved the paradox. Cytogenetic analysis following treatment with anti-$AKR^{\theta}$ in the presence of complement and subsequent T cell stimulation (Con. A) revealed a significant number of AKR cells (Barnes, Tuffrey, Bona, Evans and Ford, unpublished data). This led us to consider the existence of AKR T cells without their corresponding $AKR^{\theta}$ antigen and the possibility that these cells might be processed with $CBA^{\theta}$ antigen. If proven, this suggests that $\theta$ processing would have occurred independent of the cellular genome. In man the Lewis red cell group is acquired from a source other than the red cell or its precursor (Race and Sanger, 1958). It is conceivable that a similar process has occurred here in respect of $\theta$ processing.

Theta processing might well be a vital clue in respect of tumour immunity. In contrast to most AKR sublines, two sublines, namely AKR/Cum and AKR/RuA appear exceptional in not only being relatively resistant to lymphomata but also in having $\theta^{C3H}$ rather than $\theta^{AKR}$ (Acton *et al.*, 1973). Conceivably these two factors might be related and thus we are left with the possibility that CBA $\theta$ processing of AKR cells in the chimaeras may have been directly responsible for their tumour resistance. It is an attractive hypothesis to consider that this process of tolerance to the oncogenic Gross virus, may have been maintained and in the absence of "masking" of tumour specific sites by antibody–viral antigen complexes "normal" tumour immune mechanisms are effected. This hypothesis, however, remains to be proven.

We are grateful for the expert advice of Dr Jo Hilgers and Dr June East. The expert technical assistance of Misses Jean Kingman, Pamela Lund and Linda Dawson is acknowledged.

### REFERENCES

Acton, R. T., Blankenhorn, E. P., Douglas, T. C., Owen, R. D., Hilgers, J., Hoffman, H. A. & Boyse, E. A. (1973) AKR Mice—Genetic Variation among Sublines. *Nature, New Biol.*, **245**, 8.

Alford, C. A. Jr, Neva, F. A. & Weller, T. H. (1964) Virologic and Serologic Studies on Human Products of Conception after Maternal Rubella. *New Engl. J. Med.*, **271**, 1275.

Banks, K. L. & Henson, J. B. (1969) Glomerular Deposition of Gamma Globulin and Complement (C'3) in Equine Infectious Anaemia. *Fedn Proc.*, **28**, 752.

Barnes, R. D. (1974*a*) Leukaemia—an Immune Deficiency? *Adv. Biosci.*, **12**, 160.

Barnes, R. D. (1974*b*) The Use of Embryo Transfer and Embryo Fusion to Study the Immune Response to Virus Infection in Mice. *Proc. WHO Symp. on Infection and Immunology in Rheumatic Diseases.* Oxford: Blackwell. In the press.

Barnes, R. D. & Tuffrey, M. (1974*a*) Absence of Lymphomas in CBA Mice derived by Embryo Transfer and Born from Lymphoma-prone AKR Mice. *Eur. J. Cancer.* In the press.

Barnes, R. D. & Tuffrey, M. (1974*b*) Lymphoma Susceptibility of the AKR Mouse Strain Acquired before the Stage of Implantation. *Br. J. Cancer*, **29**, 400.

Barnes, R. D., Tuffrey, M., Drury, L. & Catty, D. (1974*a*) Unequal Rates of Cell Proliferation in Tetraparental Mouse Chimaeras Derived by Fusion of Early Embryos. *Differentiation.* In the press.

Barnes, R. D., Tuffrey, M. & Ford, C. E. (1973) Suppression of Lymphoma Development in Tetraparental AKR Mouse Chimaeras Derived from Ovum Fusion. *Nature, New Biol.*, **244**, 282.

Barnes, R. D., Tuffrey, M., Holliday, J., Hilgers, J. H. M. & Souissi, T. (1974*b*) Gross Viral Antigen in Tumour Resistant Tetraparental AKR↔CBA/H-T6 Chimaeras. *Eur. J. Cancer.* In the press.

BARNES, R. D., TUFFREY, M. & KINGMAN, J. (1972*a*) The Delay of Leukaemia in Tetraparental Ovum Fusion-derived AKR Chimaeras. *Clin. & exp. Immunol.*, **12**, 541.

BARNES, R. D., TUFFREY, M., KINGMAN, J., THORNTON, C. & TURNER, M. W. (1972*b*) The Disease of the NZB Mouse. I: Examination of Ovum Fusion Derived Tetraparental NZB : CFW Chimaeras. *Clin. & exp. Immunol.*, **11**, 605.

BONA, C. A., TUFFREY, M. & BARNES, R. D. (1974) Distribution of Theta Antigens in Ovum Fusion Derived AKR↔CBA/H-T6T6 Tetraparental Mouse Chimaeras. *Tissue Antigens*, **4**, 31.

FORD, C. E., EVANS, E. P., BURTENSHAW, M. D., CLEGG, H., BARNES, R. D. & TUFFREY, M. (1974) Marker Chromosome Analysis of Chimaeras: Dominance of AKR Mitoses in Tetraparental AKR↔CBA-T6 Mice. *Differentiation.* In the press.

FURTH, J., SEIBOLD, H. R. & RATHBONE, R. R. (1933) Experimental Studies on Lymphomatosis of Mice. *Am. J. Cancer*, **19**, 521.

GROSS, L. (1951) "Spontaneous" Leukemia Developing in C3H Mice following Inoculation, in Infancy, with AK-leukemia Extracts or AK-embryos. *Proc. Soc. exp. Biol.*, **76**, 27.

HIRSCH, M. S., ALLISON, A. C. & HARVEY, J. J. (1969) Immune Complexes in Mice Infected Neonatally with Moloney Leukaemogenic and Murine Sarcoma Viruses. *Nature, Lond.*, **223**, 739.

LILLY, F. & PINCUS, T. (1973) Genetic Control of Murine Viral Leukemogenesis. *Adv. Cancer Res.*, **17**, 231.

MARKHAM, R. V., SUTHERLAND, J. C., CIMMO, E. F., DRAKE, W. P. & MARDINEY, M. R. (1972) Immune Complexes Localized in the Renal Glomeruli of AKR Mice: The Presence of MuLV-gs1 and C-type RNA Tumour Virus gs-3 Determinants. *Rev. Eur. Étud. clin. biol.*, **XVII**, 690.

MINTZ, B. (1962) Experimental Study of the Developing Mammalian Egg: Removal of the Zona Pellucida. *Science, N.Y.*, **138**, 594.

MURPHY, F. D. (1966) Characteristic Tumors. In *Biology of the Laboratory Mouse.* Ed. E. L. Green. New York: The Blakiston Div./McGraw-Hill Book Co.

NOTKINS, A. L., MAGE, M., ASHE, W. K. & MAHAR, S. (1968) Neutralization of Sensitized Lactic Dehydrogenase Virus by Anti-gammaglobulin. *J. Immun.*, **100**, 314.

NOTKINS, A. L., MAHAR, S., SCHEELE, C. & GOFFMAN, J. (1966) Infectious Virus–antibody Complex in the Blood of Chronically Infected Mice. *J. exp. Med.*, **124**, 81.

OLDSTONE, M. B. A., AOKI, T. & DIXON, F. J. (1972) The Antibody Response of Mice to Murine Leukemia Virus in Spontaneous Infection: Absence of Classical Immunological Tolerance. *Proc. natn. Acad. Sci. U.S.A.*, **69**, 134.

OLDSTONE, M. B. A. & DIXON, F. J. (1967) Lymphocytic Choriomeningitis: Production of Antibody by "Tolerant" Infected Mice. *Science, N.Y.*, **158**, 1193.

OLDSTONE, M. B. A. & DIXON, F. J. (1968) Early Events in Allergic Encephalomyelitis. *Trans. Am. neurol. Ass.*, **93**, 257.

OLDSTONE, M. B. A. & DIXON, F. J. (1971) Lactic Dehydrogenase Virus-induced Immune Complex Type of Glomerulonephritis. *J. Immun.*, **106**, 1260.

OROSZLAN, S., FISHER, C. L., STANLEY, T. B. & GILDEN, R. V. (1970) Proteins of the Murine C-type RNA Tumour Viruses: Isolation of a Group-specific Antigen by Isoelectric Focusing. *J. gen. Virol.*, **8**, 1.

PHILLIPS, C. A., MELNICK, J. L., YOW, M. D., BAYATPOUR, M. & BURKHARDT, M. (1965) Persistence of Virus in Infants with Congenital Rubella and in Normal Infants with a History of Maternal Rubella. *J. Am. med. Ass.*, **193**, 1027.

PLOTKIN, S. A., DUDGEON, J. A. & RAMSAY, A. M. (1963) Laboratory Studies on Rubella and the Rubella Sydrome. *Br. med. J.*, ii, 1296.

PORTER, D. D. & LARSEN, A. E. (1967) Aleutian Disease of Mink: Infectious Virus–antibody Complexes in the Serum. *Proc. Soc. exp. Biol. Med.*, **126**, 680.

PORTER, D. D., LARSEN, A. E. & PORTER, H. G. (1969) The Pathogenesis of Aleutian Disease of Mink. I. *In vivo* Replication and the Host Antibody Response to Viral Antigen. *J. exp. Med.*, **130**, 575.

PORTER, D. D. & PORTER, H. G. (1971) Deposition of Immune Complexes in the Kidneys of Mice Infected with Lactic Dehydrogenase Virus. *J. Immun.*, **106**, 1264.

RACE, R. R. & SANGER, R. (1958) *Blood Groups in Man.* Springfield, Illinois: Charles C. Thomas.

TARKOWSKI, A. K. (1961) Mouse Chimaeras Developed from Fused Eggs. *Nature, Lond.*, **190**, 857.

TUFFREY, M., BARNES, R. D., EVANS, E. P. & FORD, C. E. (1973) Dominance of AKR Lymphocytes in Tetraparental AKR↔CBA-T6T6 Chimaeras. *Nature, New Biol.*, **243**, 207.

WELLER, T. H., ALFORD, C. A. JR & NEVA, F. A. (1964) Retrospective Diagnosis by Serologic Means of Congenitally Acquired Rubella Infections. *New Engl. J. Med.*, **270**, 1039.